# ﻿Review of the wolf spider genus *Acantholycosa* Dahl, 1908 from China (Araneae, Lycosidae)

**DOI:** 10.3897/zookeys.1240.146399

**Published:** 2025-06-06

**Authors:** Xiang-Yun Zhang, Zhi-Sheng Zhang, Lu-Yu Wang

**Affiliations:** 1 Key Laboratory of Eco-environments in Three Gorges Reservoir Region (Ministry of Education), School of Life Sciences, Southwest University, Chongqing 400715, China Southwest University Chongqing China

**Keywords:** Description, morphology, new record, new species, Pardosinae, taxonomy

## Abstract

The genus *Acantholycosa* Dahl, 1908 from China is reviewed, including five species: *A.aborigenica* Zyuzin & Marusik, 1988, *A.lignaria* (Clerck, 1757), *A.sterneri* (Marusik, 1993), *A.zang***sp. nov.** (♂♀) and *A.zhangi***sp. nov.** (♂♀). *Acantholycosaaborigenica* and *A.sterneri* are recorded from China for the first time. Detailed descriptions are presented, along with photos of the habitus and copulatory organs, and a distribution map.

## ﻿Introduction

*Acantholycosa* Dahl, 1908 is a relatively large genus, comprising 37 species from the Holarctic (WSC 2025). Most species are endemics, restricted to the mountainous regions of South Siberia and Far East Russia (Marusik et al. 2004; Fomichev and Omelko 2020; Fomichev 2021). The genus is relatively well studied due to the existence of one global (Marusik et al. 2004) and several regional revisions (Fomichev and Marusik 2018; Fomichev and Omelko 2020; Fomichev 2021).

In China, only two *Acantholycosa* species have been recorded: *A.baltoroi* (Caporiacco, 1935) and *A.lignaria* (Clerck, 1757). However, examination of the type specimen of *A.baltoroi* from India proved that it was a member of another wolf spider genus, *Evippa* Simon, 1882. The misidentification by Buchar (1976) – based on Roewer’s (1955) incorrect generic placement – and by the subsequent authors indicated in the synonymy of the new species, means that all records of *A.baltoroi* were misidentified, leaving *A.lignaria* as the sole species of *Acantholycosa* reported from China (Sankaran and Caleb 2023).

Based on the examination of newly collected specimens or those previously identified as *A.baltoroi* from Hebei, Inner Mongolia, Jilin, Sichuan and Xizang, we reviewed the material and found two new species: *A.zang* sp. nov. and *A.zhangi* sp. nov., two species previously unknown in China (*A.aborigenica* Zyuzin & Marusik, 1988 and *A.sterneri* (Marusik, 1993)), and the previously reported species *A.lignaria*.

## ﻿Material and methods

All specimens were preserved in 75% ethanol and examined, illustrated, photographed and measured using a Leica M205A stereomicroscope equipped with a drawing tube, a Leica DFC450 camera, and Leica Application Suite software (version 4.6). Male palps and epigynes were examined and illustrated after dissection. Epigynes were cleared in a pancreatin solution (Álvarez-Padilla and Hormiga 2007). Leg measurements are shown as: total length (femur, patella + tibia, metatarsus, tarsus). All measurements are in millimetres. Specimens examined here are deposited in the spider collection at the School of Life Sciences, Southwest University, Chongqing of China (**SWUC**).

Abbreviations used in the text: ALE, anterior lateral eye; AME, anterior median eye; C, conductor; CO, copulatory opening; E, embolus; FD, fertilization duct; Ho, hood; HS, head of spermatheca; MA, median apophysis; Pa, paleal apophysis; PLE, posterior lateral eye; PME, posterior median eye; Sb, septal base; Ss, septal stem; SS, stalk of spermatheca; St, subtegulum; TA, terminal apophysis; Te, tegulum. Terminology in the article follows Wang et al. (2025).

## ﻿Taxonomy

**Family Lycosidae Sundevall, 1833**

### ﻿Genus Acantholycosa

Taxon classificationAnimaliaAraneaeLycosidae

Dahl, 1908

8D6CB7F9-101A-5D8B-86D6-FBED563238CE

#### Type species.

*Lycosasudetica* L. Koch, 1875.

#### Diagnosis.

Members of *Acantholycosa* can be recognized by tibia I with 4, 5 or 6 pairs of ventral spines; palea modified, with a laminar or claw-like outgrowth referred to as a paleal apophysis; terminal apophysis long, with spine-like end; median apophysis with reduced apical arm (smaller than basal arm, except in *A.oligerae* Marusik, Azarkina & Koponen, 2004 and *A.petrophila* Marusik, Azarkina & Koponen, 2004); and presence of a spine-shaped or triangular outgrowth at the base of the embolus in about half of the species (Marusik et al. 2004). Most females of this genus can be recognized by 4 to 6 pairs of ventral spines on tibia I, an elongate epigyne subdivided into an atrium (basal part) and upper flat part (exception *baltoroi*-group), hoods fused or almost fused (not separated by septum) and located far from atrium, and long spermathecae.

#### Description.

Medium sized (5.86–11.19). Carapace pear-shaped, with partially visible dark lateral bands, from moderately light colored to almost black. Fovea longitudinal and brown. Cervical groove and radial furrows indistinct. Eye region black. Chelicerae light brown to dark brown, with three promarginal and three retromarginal teeth. Endites and labium light brown to dark brown, longer than wide. Sternum pale brown to dark brown, shield shaped, covered with setae. Legs yellowish brown to dark brown, with black annulations. Spination variable, tibia I with 4 to 6 pairs of ventral spines. Leg formula: 4123, 4132 or 4312. Abdomen oval, dorsum light colored to almost black, without distinct pattern. Heart mark distinct or indistinct, lanceolate, light yellow to black. Venter light brown to brown. Spinnerets pale yellow to dark brown.

**Figure 1.**
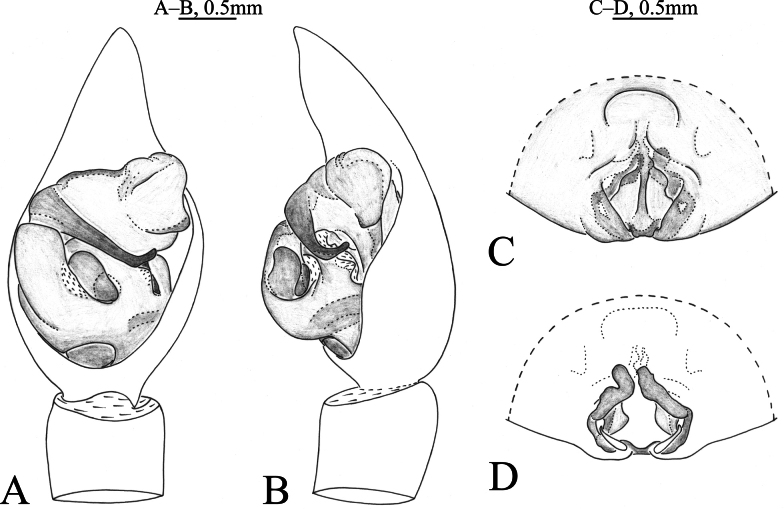
*Acantholycosaaborigenica* Zyuzin & Marusik, 1988, male (**A, B**) and female (**C, D**) **A** left male palp, ventral view **B** same, retrolateral view **C** epigyne, ventral view **D** same, dorsal view.

**Figure 2.**
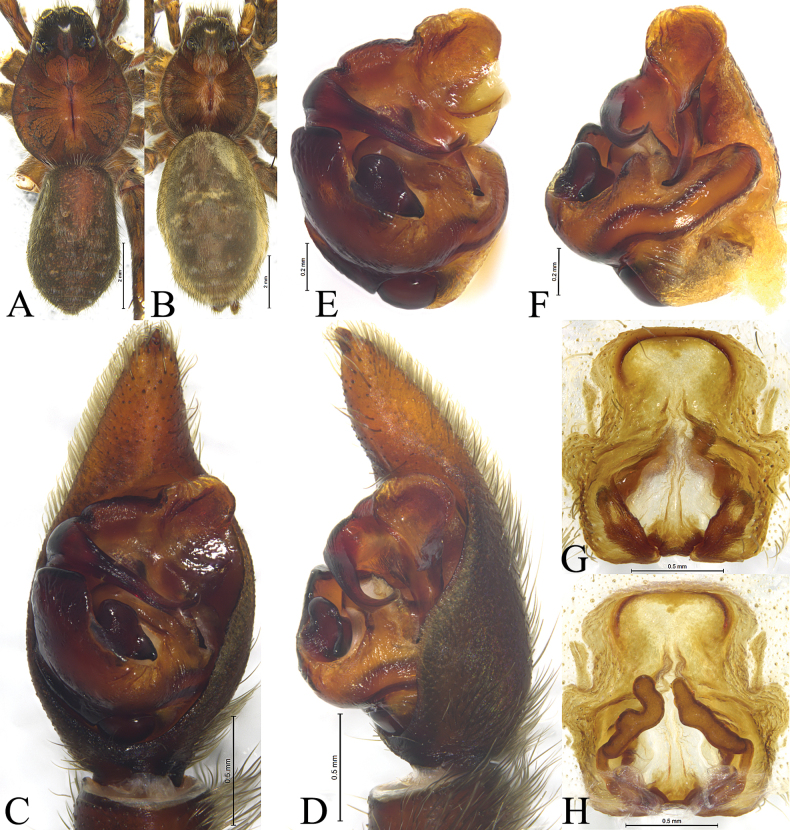
*Acantholycosaaborigenica* Zyuzin & Marusik, 1988, male (**A, C–F**) and female (**B, G, H**) **A** male habitus, dorsal view **B** female habitus, dorsal view **C** left male palp, ventral view **D** same, retrolateral view **E** palp bulb, ventral view **F** same, retrolateral view **G** epigyne, ventral view **H** same, dorsal view.

**Figure 3.**
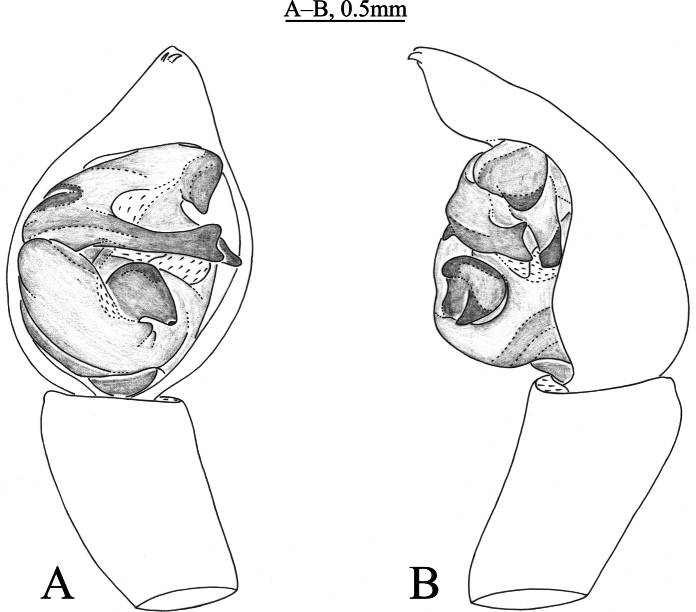
*Acantholycosalignaria* (Clerck, 1757), male **A** left male palp, ventral view **B** same, retrolateral view.

**Figure 4.**
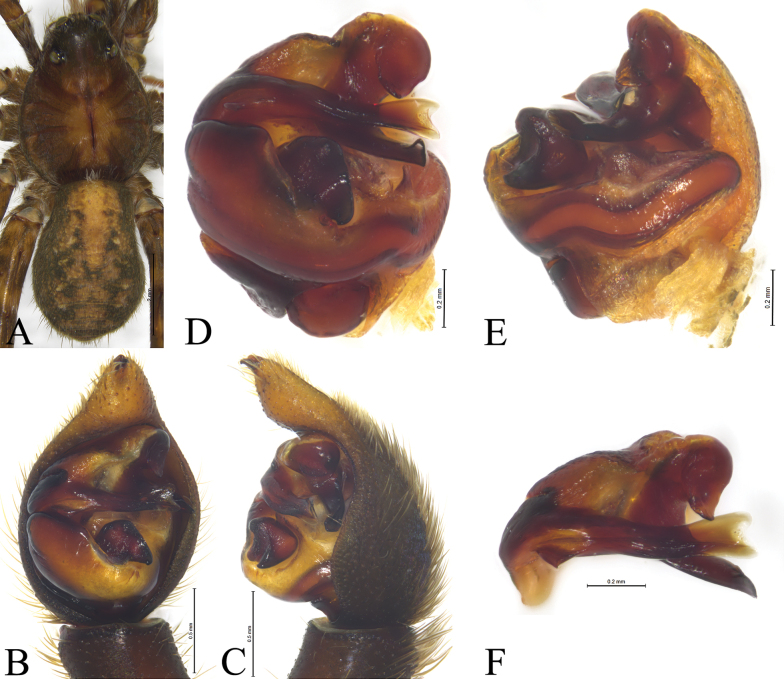
*Acantholycosalignaria* (Clerck, 1757), male **A** male habitus, dorsal view **B** left male palp, ventral view **C** same, retrolateral view **D** palp bulb, ventral view **E** same, retrolateral view **F** paleal apophysis, terminal apophysis and embolus, ventral view.

**Figure 5.**
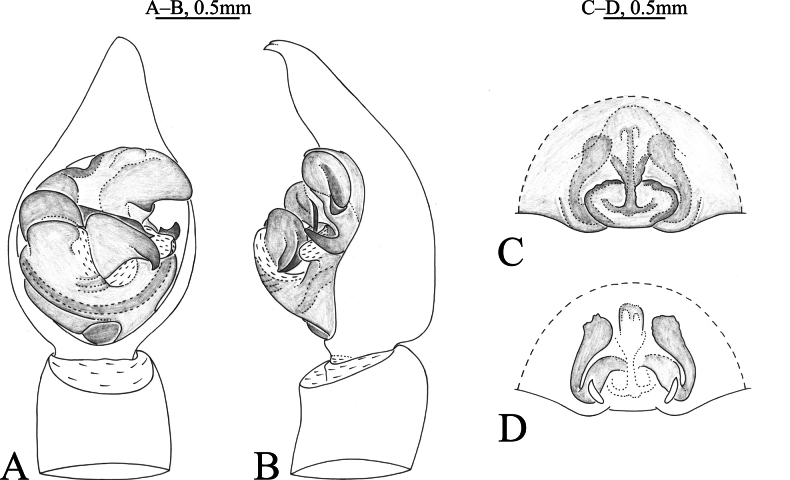
*Acantholycosasterneri* (Marusik, 1993), male (**A, B**) and female (**C, D**) **A** left male palp, ventral view **B** same, retrolateral view **C** epigyne, ventral view **D** same, dorsal view.

**Figure 6.**
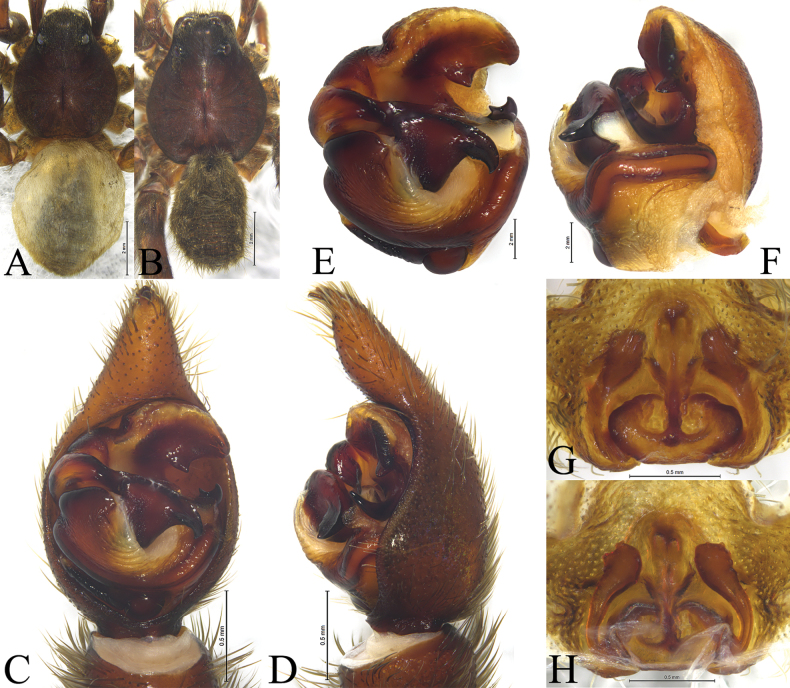
*Acantholycosasterneri* (Marusik, 1993), male (**A, C–F**) and female (**B, G, H**) **A** male habitus, dorsal view **B** female habitus, dorsal view **C** left male palp, ventral view **D** same, retrolateral view **E** palp bulb, ventral view **F** same, retrolateral view **G** epigyne, ventral view **H** same, dorsal view.

**Figure 7.**
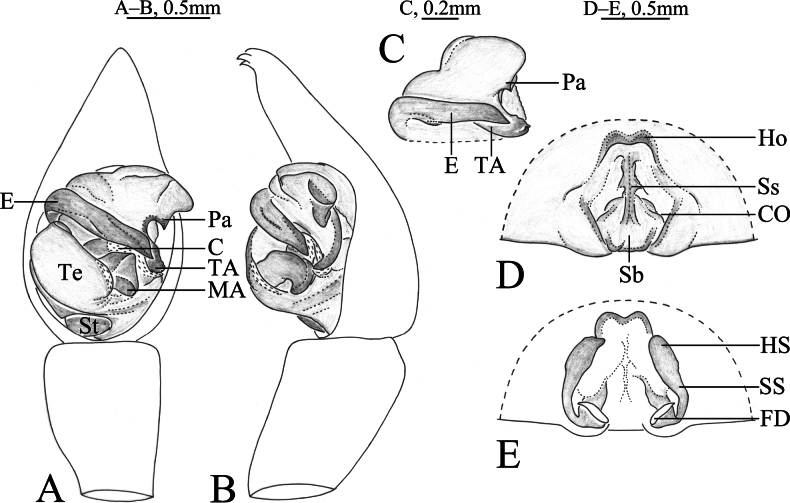
*Acantholycosazang* sp. nov., holotype male (**A, B**), paratype male (**C**) and paratype female (**D, E**) **A** left male palp, ventral view **B** same, retrolateral view **C** paleal apophysis, terminal apophysis and embolus, ventral view **D** epigyne, ventral view **E** same, dorsal view. Abbreviations: C = conductor; CO = copulatory opening; E = embolus; FD = fertilization duct; Ho = hood; HS = head of spermathecae; MA = median apophysis; Pa = paleal apophysis; Sb = septal base; Ss = septal stem; SS = stalk of spermathecae; St = subtegulum; TA = terminal apophysis; Te = tegulum.

**Figure 8.**
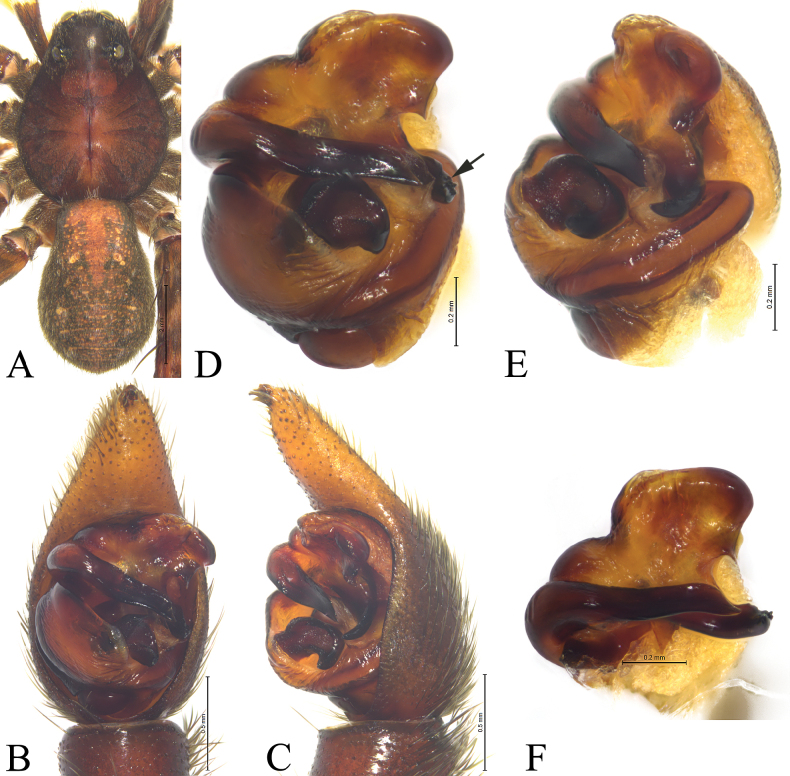
*Acantholycosazang* sp. nov., holotype male (**A–C**) and paratype male (**D–F**) **A** male habitus, dorsal view **B** left male palp, ventral view **C** same, retrolateral view **D** palp bulb, ventral view **E** same, retrolateral view **F** paleal apophysis, terminal apophysis and embolus, ventral view.

***Palp*** (Figs 1A, B, 2C–F, 3A, B, 4B–F, 5A, B, 6C–F, 7A–C, 8B–F, 10A–C, 11B–F) with uniformly brown to dark brown and droplet-shaped cymbium. Number of claws variable, from 1 to 3. Palea modified, with three conformations: plate-like outgrowth, claw- or hook-like apophysis, and small triangular outgrowth. Tip of terminal apophysis with three modifications: small spine, strong conical spine- or claw-like outgrowth, and strong conical or cylindrical outgrowth bifurcate at tip or truncate. Median apophysis with two arms: apical arm and basal arm. Apical arm usually fully or partially reduced. Embolus wide, broader in terminal half. Tip of embolus with at least four variations: truncate, widened terminally and bifurcate, sharply curved in direction of bulb apically and slightly curved beyond bulb. Basal third of embolus in majority of east Palaearctic species with modifications: small spine, big conical spine, long outgrowth subdivided apically, and flat triangular lamina. Conductor membranous.

**Figure 9.**
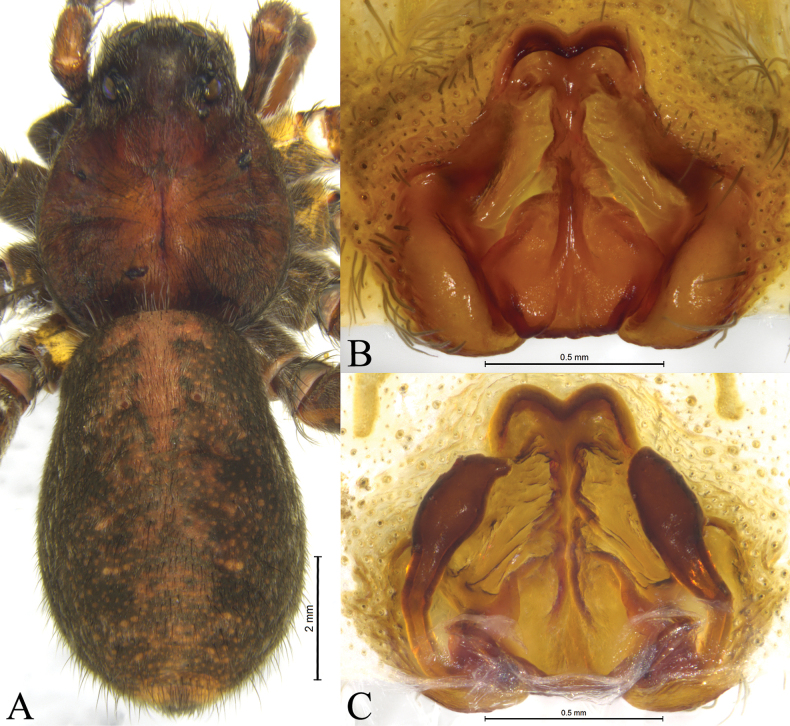
*Acantholycosazang* sp. nov., paratype female **A** female habitus, dorsal view **B** epigyne, ventral view **C** same, dorsal view.

**Figure 10.**
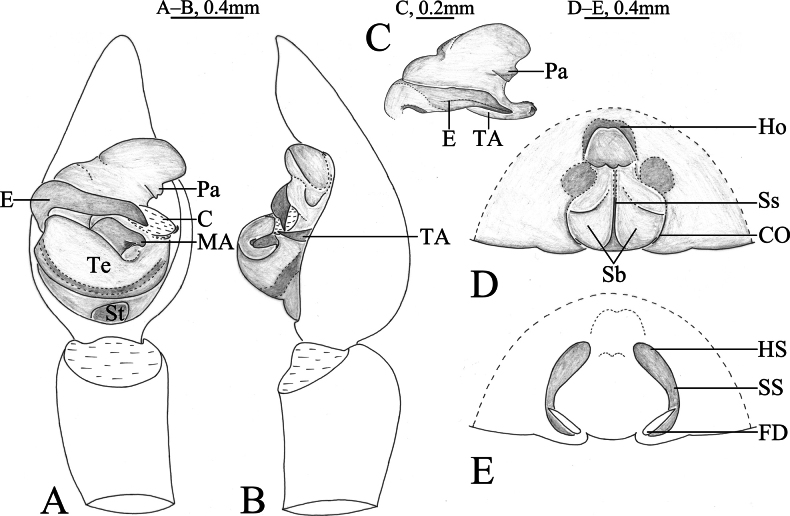
*Acantholycosazhangi* sp. nov., holotype male (**A, B**), paratype male (**C**) and paratype female (**D, E**) **A** left male palp, ventral view **B** same, retrolateral view **C** paleal apophysis, terminal apophysis and embolus, ventral view **D** epigyne, ventral view **E** same, dorsal view. Abbreviations: C = conductor; CO = copulatory opening; E = embolus; FD = fertilization duct; Ho = hood; HS = head of spermathecae; MA = median apophysis; Pa = paleal apophysis; Sb = septal base; Ss = septal stem; SS = stalk of spermathecae; St = subtegulum; TA = terminal apophysis; Te = tegulum.

***Epigyne*** (Figs 1C, D, 2G, H, 5C, D, 6G, H, 7D, E, 9B, C, 10D, E, 12B, C) with three modifications of hood: undivided, two hoods fused to some extent, or two separate hoods. Atrium well developed. In several species, septal base covering nearly almost entire atrium. Septum longer than wide, with variable base. Copulatory openings located at anterolateral or posterolateral margin of septal base. Spermathecae long, without loops or sharp turns. Heads of spermathecae with wart-like projections in many species.

#### Composition.

37 species.

#### Biology.

Almost all *Acantholycosa* species live in mountain scree (Marusik et al. 2004).

#### Distribution.

Widespread throughout the Holarctic.

### ﻿ Acantholycosa aborigenica

Taxon classificationAnimaliaAraneaeLycosidae

Zyuzin & Marusik, 1988

8EE22DEF-B0BA-5151-BB30-DAC6B58D2E0A

Figs 1 , 2 , 13

Acantholycosa sudetica Loksa, 1965: 16, fig. 23 (♀).
Pardosa aborigenica : Platnick 1993: 492.
Acantholycosa aborigenica Zyuzin & Marusik, 1988: 1083, figs 1–6 (♂♀); Marusik et al. 2004: 123, figs 108–114, 125–127, 147–151 (♂♀); Marusik and Omelko 2011: 5, figs 22, 35–36 (♂♀); Fomichev and Omelko 2020: 264, figs 31–40 (♂♀).

#### Material examined.

**China**: • 1♂ 1♀, **Inner Mongolia**, Hulun Buir, Genhe City, Hanma National Nature Reserve, Abei Forest Farm, 51°48'42"N, 122°38'47"E, elev. 1023 m, 2.07.2018, R.B. Wu leg.

#### Diagnosis.

*Acantholycosaaborigenica* is similar to *A.irinae* Fomichev & Omelko, 2020 (Fomichev and Omelko 2020, figs 1, 2, 7, 8, 13–15, 22–24, 48–49) in having the embolic tip bent anteriorly and median apophysis lacking apical arm (Figs 1A, B, 2C–F). The males of *A.aborigenica* can be distinguished from those of *A.irinae* by the paleal apophysis with broadened end ventrally (Figs 1A, 2C, E) (vs. sharp); median apophysis with triangular basal arm in ventral view (Figs 1A, 2C, E) (vs. semi-oval); absence of embolic spine near embolic base (Figs 1A, 2C, E) (vs. presence). Females can easily be distinguished from those of *A.irinae* by the septal base occupying 1/4 atrium (Figs 1C, 2G) (vs. 1/2), and septal stem reaching the apical pocket (Figs 1C, 2G) (vs. not reaching the apical pocket).

#### Description.

See Marusik et al. (2004). Habitus as shown in Fig. 2A, B, male palp as in Figs 1A, B, 2C–F, epigyne as in Figs 1C, D, 2G, H.

#### Distribution.

From Central Aimag in Mongolia to the upper reaches of the Kolyma River and south to Inner Mongolia (China).

#### Comment.

This species belongs to the *A.lignaria*-group.

### ﻿ Acantholycosa lignaria

Taxon classificationAnimaliaAraneaeLycosidae

(Clerck, 1757)

47E80A79-6157-535F-BC75-38F002393868

Figs 3 , 4 , 13

Araneus lignarius Clerck, 1757: 90, pl. 4, fig. 4 (♂♀).
Acantholycosa lignaria : Dahl 1908: 367, 369, fig. 61 (♂♀); Song et al. 1999: 316, fig. 186B (♀); Marusik et al. 2004: 119, figs 27–29, 54, 115–121 (♂♀); Almquist 2006: 184, figs 187a–h (♂♀); Marusik and Omelko 2011: 6, figs 23, 33, 34, 37 (♂♀).^1^

#### Material examined.

**China**: • 3♂, **Inner Mongolia**, Hulun Buir, Ewenki Co., Yiminhe Town, Yimin River bank, 48°36'6"N, 119°47'40"E, elev. 669 m, 26–31.07.1972, J.M. He and D.R. Yang leg. • 1♂, **Jilin Prov.**, Yanbian Pref., Antu Co., Erdaobaihe Town, 42°26'11"N, 128°6'1"E, elev. 728 m, 24.06.1979.

#### Diagnosis.

*Acantholycosalignaria* is similar to *A.zonsteini* Marusik & Omelko, 2017 (Marusik and Omelko 2017, figs 1–9) in having a rather large paleal apophysis, bilobated tip of embolus and flat and broad embolic spine (Figs 3A, B, 4B–F), but it can be differentiated by the terminal apophysis angled ventrally (Figs 3A, 4B, D, F) (vs. straight); the triangular embolic spine extending posteriorly (Figs 3A, 4B, D, F) (vs. conical spine extending prolaterally); and the subdistal part of the embolus not tapering ventrally (Figs 3A, 4B, D, F) (vs. tapering).

#### Description.

See Marusik et al (2004). Habitus as shown in Fig. 4A, male palp as in Figs 3A, B, 4B–F.

#### Distribution.

From Germany and Sweden east to Kamchatka and south to Jilin and northeastern Inner Mongolia (China).

#### Comment.

This species belongs to the *A.lignaria*-group.

### ﻿ Acantholycosa sterneri

Taxon classificationAnimaliaAraneaeLycosidae

(Marusik, 1993)

79AA48E4-994C-56C8-83ED-AA44724BF954

Figs 5 , 6 , 13

Pardosa sterneri Marusik, 1993: 77, figs 1–3 (♂).
Acantholycosa sterneri : Kronestedt and Marusik 2002: 67, figs 2, 7, 8, 12, 13, 15–18, 21, 25–27 (♂♀); Marusik et al. 2004: 113, figs 66–72 (♂♀); Marusik and Omelko 2017: 599, fig. 12 (♂).

#### Material examined.

**China**: • 1♂, **Inner Mongolia**, Chifeng City, Balinyou Co., Saihanwula National Nature Reserve, top of Hanshan Mt., 44°10'27"N, 118°44'2"E, elev. 1828 m, 27.06.2015, Z.S. Zhang and L.Y. Wang leg. • 1♀, Saihanwula National Nature Reserve, 44°18'96"N, 118°75'31"E, elev. 1941 m, 15.08.2023, S.T. Shi et al. leg. • 1♂, Balinyou Co., Wulanba Mt, 44°26'33"N, 118°42'8"E, elev. 1846 m, 11.07. 2023, K. Yu et al. leg.

#### Diagnosis.

*Acantholycosasterneri* is similar to *A.solituda* (Levi & Levi, 1951) (Kronestedt and Marusik 2002, figs 1, 3–6, 9–11, 14, 19, 20, 22–24) in having a similar conformation of the copulatory organs (Figs 5A–D, 6C–H), but it can be differentiated by the median apophysis with a reduced apical arm (Figs 5A, 6C, E) (vs. without apical arm); embolus basal part wider than apical part (Figs 5A, 6C, E) (vs. embolus basal part as wide as apical part); embolus with pointed tip ventrally (Figs 5A, 6C, E) (vs. blunt). Females can be distinguished by the septal width measuring 1.3 times its length (Figs 5C, 6G) (vs. 2.3 times its length), and spermathecae separated (Figs 5D, 6H) (vs. spermathecae close together).

#### Description.

See Kronestedt and Marusik (2002). Habitus as shown in Figs 6A, B, male palp as in Figs 5A, B, 6C–F, epigyne as in Figs 5C, D, 6G, H.

#### Distribution.

China (Inner Mongolia), Russia (South Siberia), Mongolia.

#### Comment.

This species belongs to the *A.solituda*-group.

### ﻿ Acantholycosa zang sp. nov.

Taxon classificationAnimaliaAraneaeLycosidae

ED71F0A9-F17B-53C9-AEA1-7E6579F7B3D2

https://zoobank.org/2AF6ADA8-310B-48C8-B01E-910BD979CFAE

Figs 7 , 8 , 9 , 13

Acantholycosa baltoroi : Buchar 1976: 202, figs 1–3 (♂♀); Chen et al. 1998: 72, figs 13–19 (♂♀); Song et al. 1999: 310, figs 186A, M (♂♀); Marusik et al. 2004: 112, fig. 60 (♂); Marusik and Omelko 2017: 597, fig. 10 (♂) (misidentified).

#### Type material.

• ***Holotype*** ♂ (SWUC-T-LY-25-01): **China**, **Xizang**, Nyingchi City, Zayü Co., 64^th^ km of Provincial Highway S201, 29°19'41"N, 97°8'8"E, elev. 3903 m, 25.06.2018, L.Y. Wang et al. leg. • ***Paratypes***: 3♂ 1♀ (SWUC-T-LY-25-02~05), same data as holotype • 1♂, Qamdo City, Riwoqê Co., Riwoqê Town, 31°23'26"N, 96°32'51"E, elev. 3933 m, 22.05.2017, T. Lu and Q.Y. Wang leg. • 1♂ (SWUC-T-LY-25-06), Qamdo City, Markham Co., Quzika Township, Xiaochangdu Vill., 29°11'53"N, 98°38'42"E, elev. 3496 m, 11.05.2017, T. Lu and Z.S. Wu leg. • 2♂ (SWUC-T-LY-25-07~08), Nyingchi City, Zayü Co., Guyu Township, 29°5'32"N, 97°17'13"E, elev. 3196 m, valley scrub, 25.05.2019, L.Y. Wang et al. leg. • 2♂ (SWUC-T-LY-25-09~10), **Sichuan Prov.**, Garze Pref., Dege Co., Que’er Mt., Wudaoban, 31°56'33"N, 98°55'1"E, elev. 4707 m, 18.06.2016, T. Lu et al. leg. • 7♂ 3♀ (SWUC-T-LY-25-11~20), Garze Pref., Daocheng Co., Haizi Mt., Yunsecuo River bank, 29°27'4"N, 100°11'30"E, elev. 4615 m, 12.06.2016, T. Lu et al. leg.

#### Etymology.

The specific name comes from the Chinese word “*zang*”, the name of one of the Chinese ethnic minorities that mainly live in Xizang and Sichuan provinces of China; noun in apposition.

#### Diagnosis.

**Figure 11.**
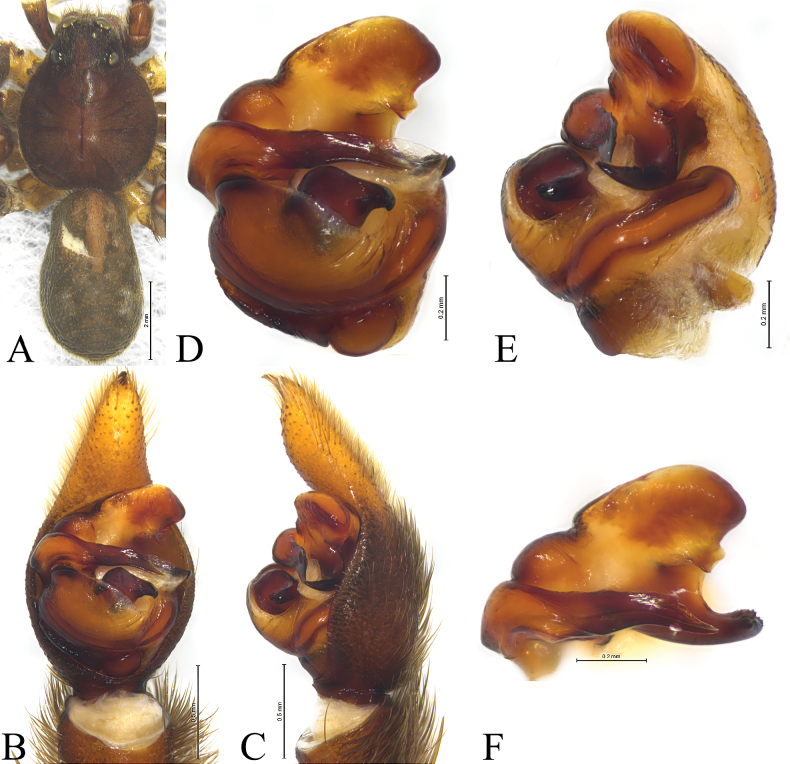
*Acantholycosazhangi* sp. nov., holotype male (**A–C**) and paratype male (**D–F**) **A** male habitus, dorsal view **B** left male palp, ventral view **C** same, retrolateral view **D** right palp bulb, ventral view (mirror flip) **E** same, retrolateral view (mirror flip) **F** paleal apophysis, terminal apophysis and embolus, ventral view.

**Figure 12.**
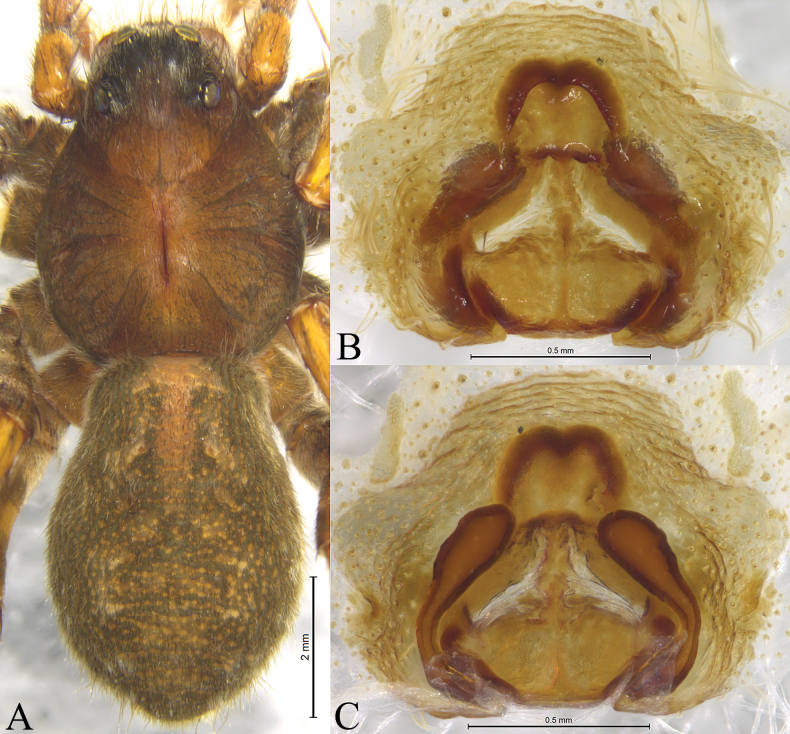
*Acantholycosazhangi* sp. nov., paratype female **A** female habitus, dorsal view **B** epigyne, ventral view **C** same, dorsal view.

This new species can be separated from other *Acantholycosa* species by the lanceolate end and retrolateral serrate margin of the terminal apophysis ventrally (Fig. 8D, arrow). The new species is most similar to *A.zhangi* sp. nov. in having a similar conformation of the copulatory organs (Figs 7A–E, 8B–F, 9B, C cf. Figs 10A–E, 11B–F, 12B, C), but it can be differentiated by the triangular paleal apophysis with a pointed tip in ventral view (Figs 7A, C, 8B, D, F) (vs. rectangular with a wavy tip; Figs 10A, C, 11B, D, F); the median apophysis axe shaped without spine (Figs 7A, 8B, D) (vs. rectangular with hook-shaped basal arm and triangular spine; Figs 10A, 11B, D); embolus with subparallel margins abruptly tapering at tip (Figs 7A, C, 8B, D, F) (vs. tapering from base to tip, slightly curved towards tip; Figs 10A, C, 11B, D, F). Females can be distinguished by having an epigyne with a pair of distinct hoods (Figs 7D, 9B) (vs. a pair of less pronounced hoods; Figs 10D, 12B); width of hoods equal to septal posterior width (Figs 7D, 9B) (vs. one-third septal posterior width; Figs 10D, 12B); septal base hexagonal (Figs 7D, 9B) (vs. trapezoidal; Figs 10D, 12B); spermathecal heads with wart-like projections (Figs 7E, 9C) (vs. smooth; Figs 10E, 12C).

#### Description.

**Male holotype** (Fig. 8A) total length 9.23. Carapace 4.87 long, 3.91 wide; opisthosoma 4.49 long, 2.99 wide. Carapace dark brown. Eye sizes and interdistances: AME 0.15, ALE 0.15, PME 0.48, PLE 0.42; AME–AME 0.17, AME–ALE 0.10, PME–PME 0.52, PME–PLE 0.55. Clypeus 0.3 high. Leg measurements: I 14.82 (3.83, 5.06, 3.88, 2.05); II 14.71 (3.99, 4.84, 3.85, 2.03); III 14.46 (3.62, 4.16, 4.56, 2.12); IV 19.73 (4.67, 5.69, 6.59, 2.78). Femur I with 5 dorsal and 3 prolateral spines; tibia I with 2 prolateral, 2 retrolateral and 8 ventral spines; metatarsus I with 5 prolateral, 5 retrolateral and 3 ventral spines.

***Palp*** (Figs 7A–C, 8B–F). Cymbium droplet-shaped, with two claws and black setae. Paleal apophysis triangular with acuminate tip. Terminal apophysis with lanceolate end and retrolateral serrate margin in ventral view. Median apophysis with concave fold dorsally and serrated margins, no apical arm. Embolus 5.5 times as long as wide, uniform, tapering abruptly at tip and without basal spine.

**Female paratype** (SWUC-T-LY-25-02, Fig. 9A) total length 11.19. Carapace 5.18 long, 4.12 wide; opisthosoma 6.45 long, 4.35 wide. Same as in male. Eye sizes and interdistances: AME 0.17, ALE 0.17, PME 0.51, PLE 0.41; AME–AME 0.24, AME–ALE 0.13, PME–PME 0.58, PME–PLE 0.64. Clypeus height 0.29. Leg measurements: I 15.33 (4.16, 5.41, 3.58, 2.18); II 15.02 (4.05, 5.12, 3.67, 2.18); III 16.40 (4.02, 5.13, 4.67, 2.58); IV 20.47 (4.98, 5.99, 6.73, 2.77). Femur I with 5 dorsal and 2 prolateral spines; tibia I with 2 prolateral, 2 retrolateral and 8 ventral spines; metatarsus I with 4 prolateral, 4 retrolateral and 3 ventral spines.

***Epigyne*** (Figs 7D, E, 9B, C). Hoods distinct, clearly separated from each other. Atrium as long as wide. Septum with distinct stem and hexagonal base equal in length and width. Width of hoods equal to septal posterior width. Copulatory openings located at anterolateral margin of septal base. Spermathecae heads oval with small wart-like projections. Spermathecal stalks slightly curved. Fertilization ducts slender, width between two ducts longer than length of duct.

#### Distribution.

China (Xizang, Sichuan), Nepal.

#### Comment.

This species belongs to the *A.solituda*-group.

#### Remark.

Although the specimens of *A.baltoroi* from Nepal were not examined, it is clear from the descriptions in Buchar (1976), Marusik et al (2004), Marusik and Omelko (2017) that *A.baltoroi* from Nepal is identical to *A.zang* sp. nov.

### ﻿ Acantholycosa zhangi sp. nov.

Taxon classificationAnimaliaAraneaeLycosidae

690F63FB-226E-5481-8239-17AE20857EC4

https://zoobank.org/FD58FC89-BDA4-4248-87DB-8153F5D1FC9F

**Figure 13.**
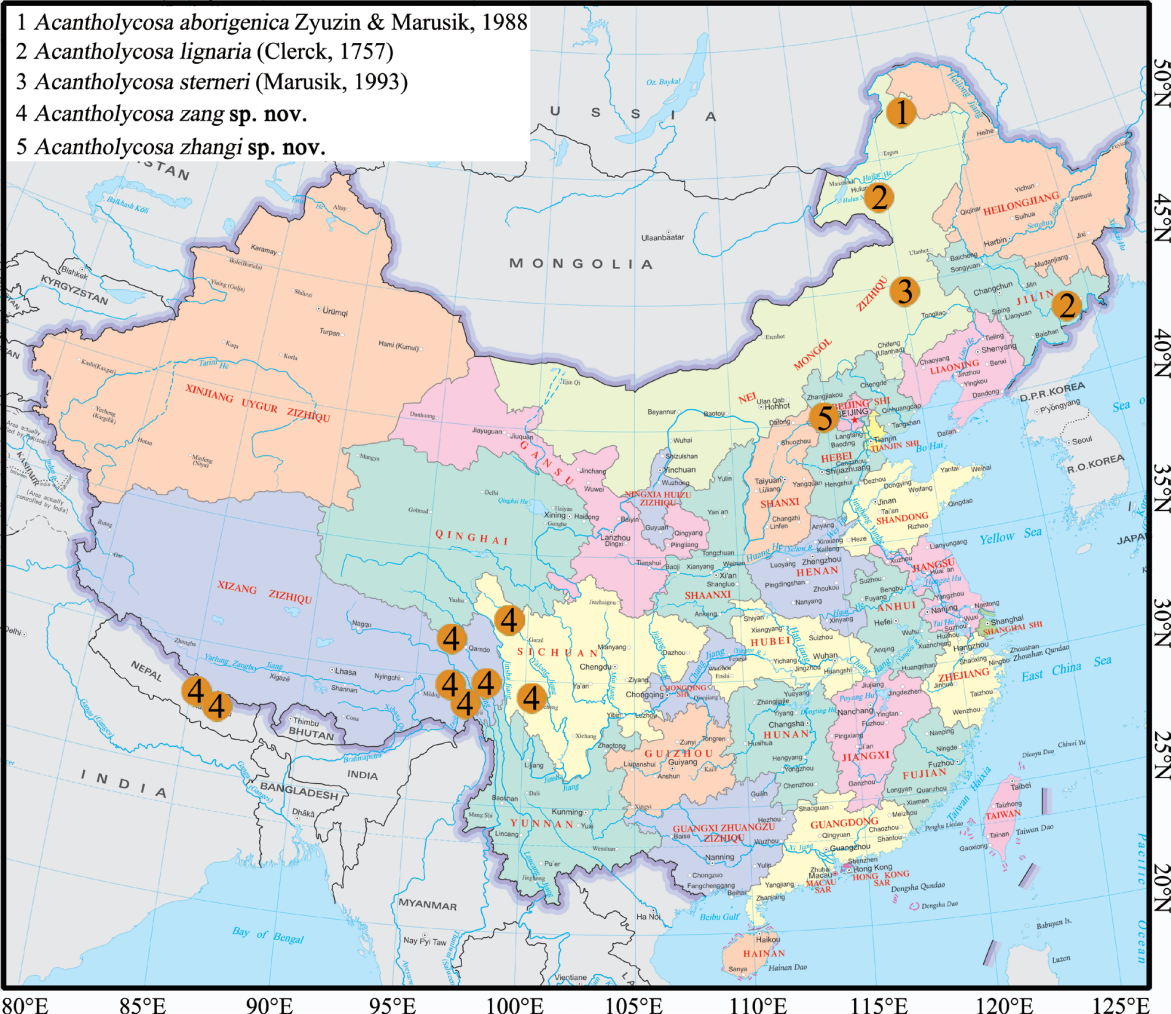
Distribution of *Acantholycosa* species in China.

Figs 10 , 11 , 12 , 13

Acantholycosa baltoroi : Song et al. 2001: 226, figs 138A–D (♂♀); Zhang et al. 2022: 119, figs 83A–J (♂♀). (misidentified).

#### Type material.

• ***Holotype*** ♂ (SWUC-T-LY-26-01) and ***paratype*** ♀ (SWUC-T-LY-26-02): **China, Hebei Prov.**, Zhangjiakou City, Yu Co., Xiaowutai Nature Reserve, Jinhekou, 39°56'17"N, 114°58'4"E, elev. 1334 m, 1.07.2012, F. Zhang leg.

#### Etymology.

The specific name comes from the family name of Prof. Feng Zhang of Hebei University (Hebei, China), who collected this new species.

#### Diagnosis.

The new species is similar to *A.zang* sp. nov. in having similar conformation of copulatory organs (Figs 10A–E, 11B–F, 12B, C; 7A–E, 8B–F, 9B, C), but differs by paleal apophysis rectangular with wavy tip in ventral view (Figs 10A, C, 11B, D, F) (vs. triangular with pointed tip; Figs 7A, C, 8B, D, F); median apophysis rectangular with hook-shaped basal arm and triangular spine (Figs 10A, 11B, D) (vs. axe shaped without spine; Figs 7A, 8B, D); embolus gradually tapering from base to tip and slightly curved (Figs 10A, C, 11B, D, F) (vs. with parallel margins except for tip suddenly tapering; Figs 7A, C, 8B, D, F). Females can be distinguished by two less pronounced (Figs 10D, 12B) (vs. distinct hoods, Figs 7D, 9B); width of hoods one-third septal posterior width (Figs 10D, 12B) (vs. width of hoods equal to septal posterior width; Figs 7D, 9B); septal base trapezoidal (Figs 10D, 12B) (vs. base hexagonal without spine; Figs 7D, 9B); spermathecal heads smooth (Figs 10E, 12C) (vs. heads with wart-like projections; Figs 7E, 9C).

#### Description.

**Male holotype** (Fig. 11A) total length 9.47. Carapace 4.78 long, 3.86 wide; opisthosoma 4.61 long, 2.78 wide. Carapace black brown with dark margins. Eye sizes and interdistances: AME 0.16, ALE 0.14, PME 0.50, PLE 0.35; AME–AME 0.14, AME–ALE 0.10, PME–PME 0.45, PME–PLE 0.62. Clypeus height 0.29. Leg measurements: I 14.56 (3.80, 5.20, 3.67, 1.89); II 14.29 (3.53, 5.08, 3.70, 1.98); III 14.16 (3.48, 4.46, 4.22, 2.00); IV 18.94 (4.47, 5.58, 6.26, 2.63). Femur I with 5 dorsal and 2 prolateral spines; tibia I with 2 dorsal, 1 prolateral, 1 retrolateral and 8 ventral spines; metatarsus I with 2 dorsal, 4 prolateral, 5 retrolateral and 3 ventral spines.

***Palp*** (Figs 10A–C, 11B–F). Cymbium with dense black setae and one claw. Paleal apophysis rectangular with wavy end. Terminal apophysis with a hook-shaped tip in retrolateral view. Median apophysis rectangular with hook-shaped basal arm and triangular spine. Embolus originating at 9 o’clock position, broad and almost straight, without basal spine, tapering from base to tip; distal end of embolus slightly curved, pointing posteriorly.

**Female paratype** (Fig. 12A) total length 10.33. Carapace 5.16 long, 3.91 wide; opisthosoma 5.43 long, 3.72 wide. Eye sizes and interdistances: AME 0.16, ALE 0.15, PME 0.50, PLE 0.41; AME–AME 0.17, AME–ALE 0.10, PME–PME 0.49, PME–PLE 0.59. Clypeus 0.54 high. Leg measurements: I 14.89 (3.92, 5.47, 3.54, 1.96); II 14.68 (4.04, 5.06, 3.58, 2.00); III 14.73 (3.88, 4.57, 4.31, 1.97); IV 20.63 (5.03, 6.18, 6.55, 2.87). Femur I with 5 dorsal and 2 prolateral spines; tibia I with 2 prolateral, 2 retrolateral and 8 ventral spines; metatarsus I with 5 prolateral, 4 retrolateral and 3 ventral spines. Except for the lighter body color, the other features are the same as those of the males.

***Epigyne*** (Figs 10D, E, 12B, C). Two hoods less pronounced and shallow. Atrium rhomboid. Septum with trapeziodal base. Width of hoods one-third septal posterior width. Spermathecal heads clavate and smooth. Spermathecal stalks slightly curved. Fertilization ducts extending antero-laterally.

#### Distribution.

China (Hebei).

#### Comment.

This species belongs to the *A.solituda*-group.

#### Remarks.

Following the taxonomic revision by Sankaran and Caleb (2023) that transferred *Acantholycosabaltoroi* to the genus *Evippa* Simon, 1882 (currently recognized as *E.baltoroi*), and the subsequent recognition that all previous records of *A.baltoroi* represented misidentifications, the former “*A.baltoroi*-group” requires nomenclatural adjustment. We hereby propose the reclassification of this species group as the *Acantholycosasolituda*-group, based on taxonomic priority. In addition, because of the closer morphological similarity between *A.lignaria* and *A.zonsteini*, we reclassify *A.zonsteini* along with the similar species *A.levinae* Marusik, Azarkina & Koponen, 2004 into the *A.lignaria*-group. Therefore, the *A.solituda*-group comprises the following valid species: *A.sergeevi* Fomichev, 2021, *A.solituda*, *A.sterneri*, and the two newly described taxa (*A.zang* sp. nov. and *A.zhangi* sp. nov.).

## Supplementary Material

XML Treatment for Acantholycosa

XML Treatment for Acantholycosa aborigenica

XML Treatment for Acantholycosa lignaria

XML Treatment for Acantholycosa sterneri

XML Treatment for Acantholycosa zang

XML Treatment for Acantholycosa zhangi

## Footnotes

For complete list of 26 taxonomic references see WSC (2025).

## ﻿Additional information

### Author ORCIDs

Xiang-Yun Zhang https://orcid.org/0009-0007-1627-3360

Zhi-Sheng Zhang https://orcid.org/0000-0002-9304-1789

Lu-Yu Wang https://orcid.org/0000-0002-5250-3473

### Data availability

All of the data that support the findings of this study are available in the main text.
